# A novel in situ simulation framework for introduction of a new technology: the 3-Act-3-Debrief model

**DOI:** 10.1186/s41077-020-00145-x

**Published:** 2020-09-25

**Authors:** Lisa T. Barker, William F. Bond, Andrew L. Vincent, Kimberly L. Cooley, Jeremy S. McGarvey, John A. Vozenilek, Emilie S. Powell

**Affiliations:** 1 Jump Simulation (an OSF Healthcare and University of Illinois College of Medicine at Peoria Collaboration), OSF HealthCare and University of Illinois College of Medicine, 1306 N. Berkeley Avenue, Peoria, IL USA; 2 Department of Emergency Medicine, OSF HealthCare, University of Illinois College of Medicine, Peoria, USA; 3 Department of Emergency Medicine, Northwestern Memorial Hospital, Northwestern University Feinberg School of Medicine, Chicago, USA

**Keywords:** Healthcare simulation, Telehealth, Debriefing, Sepsis, Interprofessional simulations, Health information technology, Implementation research

## Abstract

**Background:**

New technologies for clinical staff are typically introduced via an “in-service” that focuses on knowledge and technical skill. Successful adoption of new healthcare technologies is influenced by multiple other factors as described by the Consolidated Framework in Implementation Research (CFIR). A simulation-based introduction to new technologies provides opportunity to intentionally address specific factors that influence adoption.

**Methods:**

The new technology proposed for adoption was a telehealth cart that provided direct video communication with electronic intensive care unit (eICU) staff for a rural Emergency Department (ED). A novel 3-Act-3-Debrief in situ simulation structure was created to target predictive constructs from the CFIR and connect debriefing to specific workflows. The structure and content of the simulation in relation to the framework is described. Participants completed surveys pre-simulation/post-simulation to measure change in their readiness to adopt the new technology.

**Results:**

The scenario was designed and pilot tested before implementation at two rural EDs. There were 60 interprofessional participants across the 2 sites, with 58 pre-simulation and 59 post-simulation surveys completed. The post-simulation mean ratings for each readiness measure (feasibility, quality, resource availability, role clarity, staff receptiveness, and tech usability) increased significantly as a result of the simulation experience.

**Conclusions:**

A novel 3-stage simulation-debriefing structure positively targets factors influencing the adoption of new healthcare technologies.

## Introduction

Healthcare simulation has served a variety of functions in support of patient safety including latent threat identification [1–3] training for high-criticality/low-frequency events [4], and improving teamwork, [5–7] invasive procedural safety [8], and critical care skills [9]. Less is known about the influence of simulation on the implementation of new technologies in the healthcare setting. While there has been a call to more consistently utilize clinical simulation for testing usability of health information technologies (HIT) [10], subsequent research has focused on the use of simulation for training in preparation for a software HIT implementation [11].

**Fig. 1:**
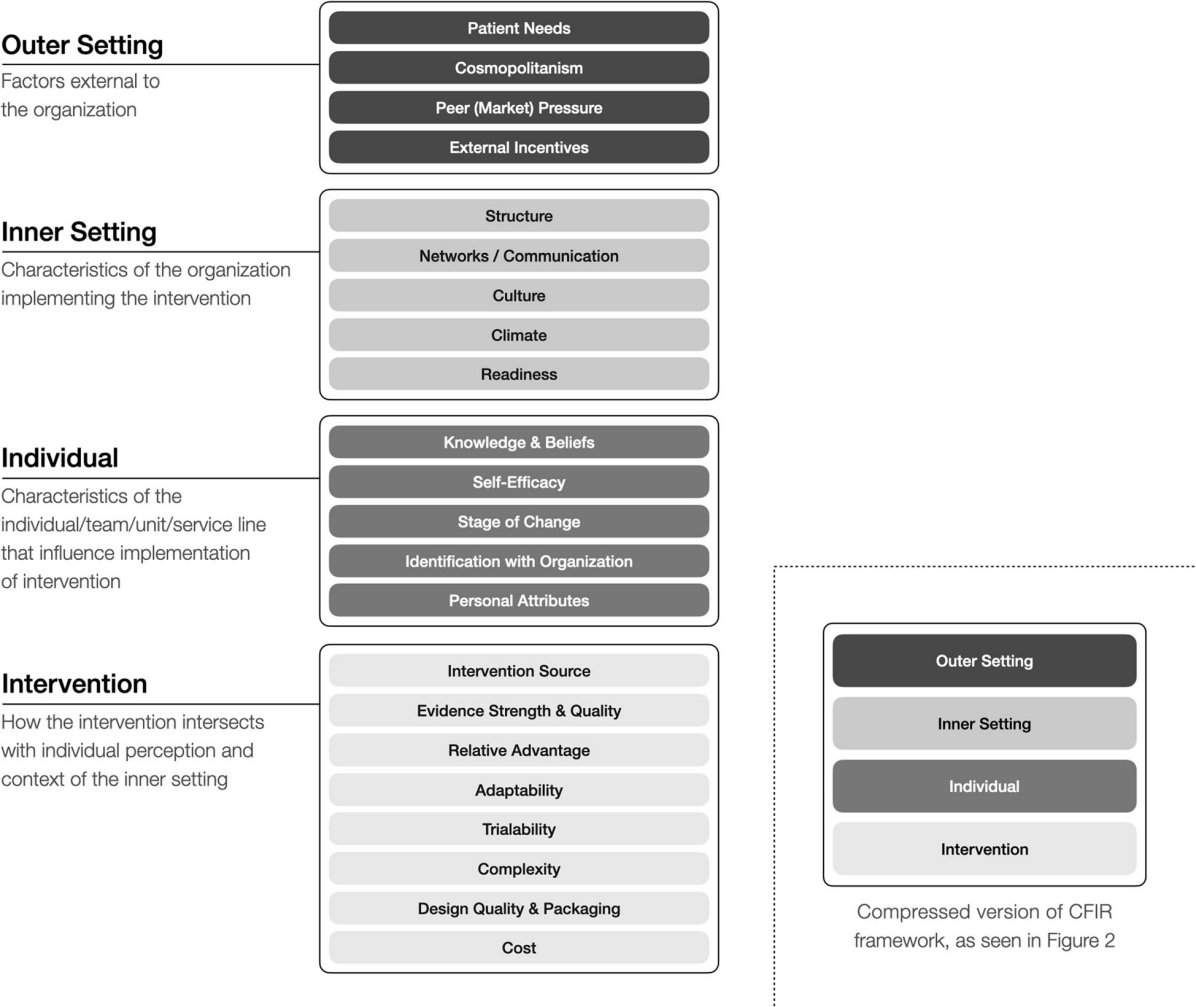
Four CFIR domains and their constructs. Complete list of the constructs within the four domains that may be influenced by simulation-based training

Predictors of technology adoption across industries have most often been studied using variations of the Technology Acceptance Model (TAM) [12], which defines acceptance as intent to use primarily based on perceived usefulness and ease-of-use [13]. However, the TAM’s fit with respect to HIT adoption has been questioned [14]. Recent studies in implementation science around clinician healthcare behaviors suggest that knowledge, while a pre-requisite for action is less correlated with simulation-based behavior change than individual beliefs around risk perception, self-efficacy, and intent to implement [15–17]. Given the complexity of change implementation in the healthcare environment, a Consolidated Framework for Implementation Research (CFIR) was proposed in 2009 [18]. This framework synthesized available implementation models with a primary focus on health services. The CFIR divides the factors that influence uptake of a new health services intervention into five domains: *intervention* characteristics, *outer setting, inner setting*, characteristics of *individuals*, and implementation *process*. Each of these domains encompasses multiple constructs. Figure 1 lists the constructs of the first four domains; all of which provide the context for the implementation process.

In situ simulation affords a unique opportunity to address these domains due to its ability to explore individual use of a HIT intervention within the settings and processes of the system. Debriefing, considered to be the phase of simulation where primary learning and reflection occurs [19, 20], also represents an opportunity to explore perceived barriers to HIT use and address them in a social context. The closest analogs in the literature are those in situ simulation efforts that describe the testing of new spaces to detect operational and safety issues [2, 21–23].

In this report, we describe a “3-Act-3-Debrief” in situ simulation structure designed to specifically support implementation of a new HIT process and technology—the use of a telemedicine cart in the care of patients with severe sepsis or septic shock in a rural emergency department (ED). While the success of “in-simulation” debriefing triggered by learner errors has been reported previously [24, 25], division of a single-simulated patient encounter into distinct phases that target HIT implementation factors is a novel approach.

The simulated encounter we describe was created as a component of a larger study evaluating the impact of telehealth use in the care of septic patients in two rural emergency departments within a 13-hospital healthcare system. The primary focus of this report is to describe a novel simulation structure that supports implementation of new healthcare technologies. As supporting evidence, the impact of the simulation utilizing the 3-Act-3-Debrief structure was measured by participants’ change in individual-related HIT implementation factors from the CFIR: perceived ease of use, feasibility, role clarity, and staff receptiveness.

## Methods

### HIT context

The specific HIT being introduced was a mobile telehealth cart that provided video teleconferencing capability between the ED and the eICU within the hospital system’s tertiary care center. The eICU is staffed 24 hours a day by critical care nurses; an intensivist physician is also present overnight to provide support for the eICUs across the healthcare system. Prior to cart introduction, the eICU critical care nursing staff interacted with ED staff solely through telephone contact, and there was no direct eICU interaction with ED patients.

The telehealth carts to be deployed in these EDs were to connect local ED nurses with the electronic intensive care unit (eICU) at the system’s tertiary care site as well as provide the option of video-based monitoring of patients with severe sepsis or septic shock.

### Scenario design

The scenario was designed to introduce the HIT in a manner that targeted simulation-accessible constructs within the domains of the CFIR: (1) patient needs, (2) structural characteristics, (3) networks and communications, (4) implementation climate, (5) complexity, (6) relative advantage, (7) knowledge and beliefs, and (8) self-efficacy in use of the intervention as perceived by staff members [18].

**Fig. 2:**
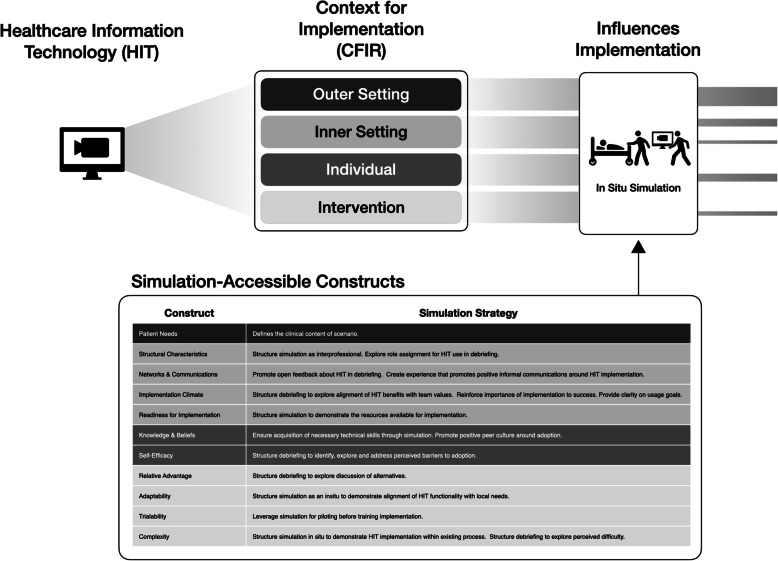
Simulation-accessible CFIR constructs. Outline of design strategies for simulations targeting HIT implementation

As shown in Fig. 2, not all constructs within the framework are amenable to simulation-based support. Within the **outer setting** domain, it is the *patient needs* construct that defines the clinical content. In our context, the treatment of sepsis was a strategic target based on sepsis bundle [26] compliance and mortality rates below desired targets. The **inner setting** constructs reflect the relevant characteristics of the organization where the HIT is being implemented. Simulation cannot directly impact the existing *structural characteristics*, which includes ‘internal division of labor’, nor *networks and communications*, which refers to existing modes of intraorganizational connection and communication. However, by designing the simulation to be interprofessional and in situ, the concepts of team roles and communication processes could be intentionally explored in the debriefings as the HIT was introduced in its real-world work team context.

Simulation experiences can more directly target the domains of *intervention characteristics* (*complexity, relative advantage*), and *characteristics of individuals* (*knowledge and beliefs, self-efficacy*) as well as the third *inner setting* construct of *implementation climate* because they all are influenced by the interactions of the individual, team, and/or unit with the HIT in the work context. For this project, the simulation was designed to provide the opportunity to train on functionality while directly using the telehealth cart in all relevant portions of workflow. The in situ simulation provides the opportunity for staff to directly experience the relative ease of deployment and where the technology could provide added benefit for patient care.

Prior to implementation, the simulation session was tested at the healthcare system’s simulation center with participation from Simulation Operations Specialists, SPs, ED physicians, nurses from the rural sites, telehealth on-site support team, and eICU nurses participating via the cart as they would during in situ simulations. This helped ensure that insights into the new process from all relevant clinical team members could be included in the scenario construction.

Initial pilot testing with ED clinical staff used a traditional scenario design—a simulated patient presenting in septic shock. This approach did not trigger consideration of the telehealth cart despite targeted briefing. Though the constructs were explored in a conversational structure for debriefing [27] the single-stage simulation encounter failed to provide opportunity to experience the HIT’s intersection with constructs of *intervention complexity*, *relative advantage*, *knowledge* *and beliefs *nor *self-efficacy*.

**Fig. 3:**
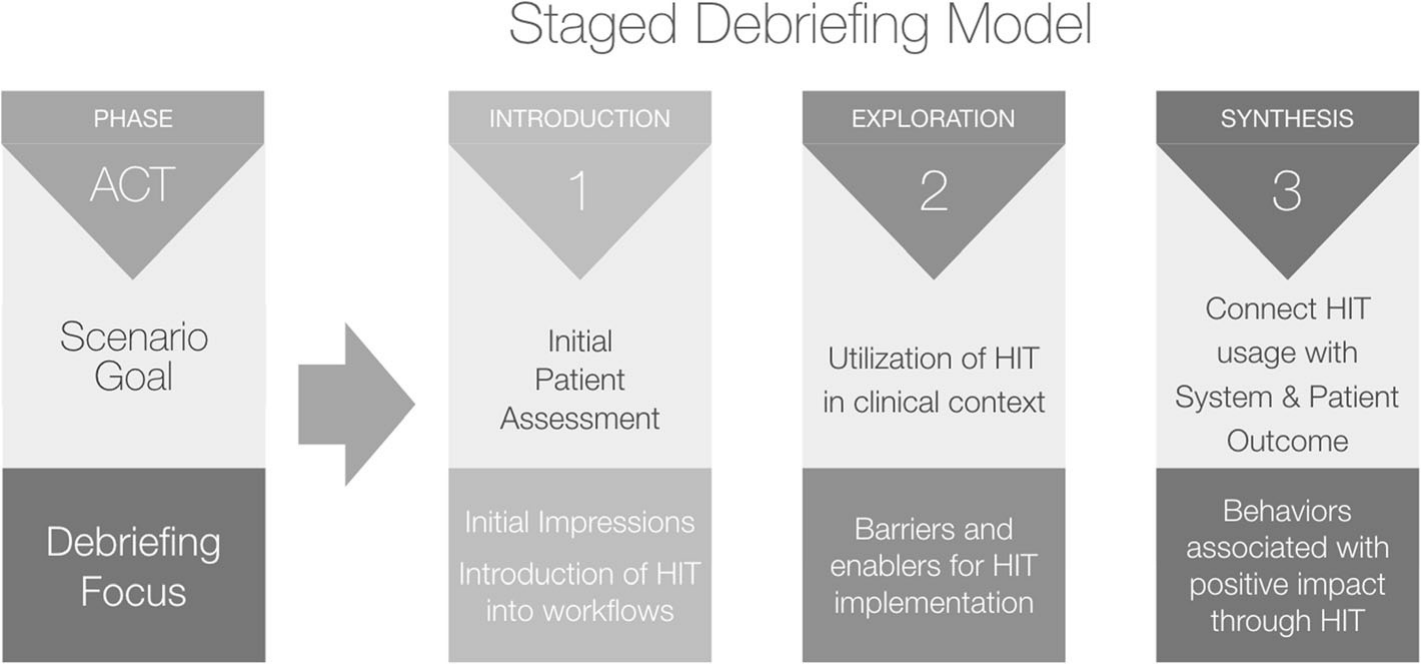
The 3-Act-3-Debrief model for HIT implementation. Three-stage framework for structuring an in situ interprofessional simulation to introduce a health information technology (HIT)

To facilitate the opportunity for more deliberate reflection and exploration of the targeted CFIR factors in the context of ED workflow, the presentation and clinical progression of the patient was subsequently divided into three “acts”, each with its own debrief (Fig. 3). The objectives in the scenario included both HIT implementation and clinical targets. Clinical content for the sepsis patient presentation and progression was based on Centers for Medicare and Medicaid Service’s (CMS) definitions and bundles of care for severe sepsis and septic shock [26] so that the team would need to progress through the full course of care during the encounter. A standardized patient (SP) was roomed as a new ED patient, initially hemodynamically stable, with progressive deterioration to begin each subsequent act. Telehealth staff, who were remote at the other end of the telehealth connection, engaged as scripted participants.

The final content summary of each act, as structured for both ED and telehealth teams, is outlined in Table 1. Corresponding debriefing around the HIT as mapped to the targeted implementation constructs is summarized in Table 2.

**Table 1:** Simulation scenario summary. Expected activities to be completed by the ED teams and embedded eICU staff during the in situ simulation

Stage	Time (min)	ED team	Telehealth
Act One	5–10	1. At bedside: full team 2. MD–perform H&P 3. RN/tech–pt on monitor, draw labs, IV start	1. Monitor cart Off 2. Brief introduction to staff when cart turned on for practice (in debrief) 3. Identify ED room number 4. No clinical discussions
Transition: *Pause for debriefing after sepsis workup initiated*			
Act Two	5–10	1. Telehealth calls bedside RN alerting him/her to BPA firing 2. Team pulls cart to just outside of room, turns it on 3. Clinical introductions 4. Reviews bundle with telehealth, additional orders/interventions	1. Telehealth nurse calls bedside nurse to alert him/her that sepsis BPA has fired on patient 2. Clinical introductions 3. Ask about bundle elements (eICU kept aware via Skype technology^24^ 4. “What sources of infection have you considered?” 5. Recommend 30 cc/kg amount which is approximately 3 L for the 100 kg SP
Transition: *Pause for debriefing after bundle elements addressed by telehealth team*			
Act Three	5–10	1. Telehealth notifies bedside RN (or MD) of patient status change–2 hours has elapsed 2. Team returns to room 3. Ensures 30 mL/kg IVF given 4. Starts pressor support 5. Arranges transfer/admission to ICU 6. Focused clinical exam	1. Verify bundle elements as needed 2. Verify classification of patient as septic shock 3. If patient admitted kept in regional ICU, emphasize eICU presence 4. Repeat lactate?
Transition: *End of case triggered by pressor support started, disposition arranged, 6 h our bundle elements addressed by telehealth team*

**Table 2:** HIT debriefing for implementation using the Consolidated Framework for Implementation Research (CFIR) Framework

	***Goal***	Debriefing outline	CFIR domain [***construct***]
**Act One**	*Introduce HIT in the context of usual workflow*	Ensure shared mental model of septic patient	**Outer setting** [*Patient needs*]
		For patients at risk for deterioration, telehealth can help team observe the patient using the cart. What is the reality of the workflow at this point?	**Inner setting** [*Structural Characteristics, Implementation Climate*]
		Explore roles—who would set up the cart?	**Intervention** [*Complexity*]
		Have participants demonstrate physical maneuvers of cart	**Intervention** [*Complexity*]**Individuals** [*Knowledge and Beliefs*]
		Telehealth RN (in person) demonstrates activating cart—introduces responding eICU nurse	**Intervention** [*Complexity*]**Inner setting** [*Networks and Communications*]
		Learners demonstrate activating cart	**Intervention** [*Complexity*]**Individuals** [*Self-Efficacy*]
		Learners interact with telehealth nurse via cart	**Intervention** [*Complexity*]
**Act Two**	*Identify anticipated barriers to* *HIT use*	Ensure shared mental model of severe sepsis	**Outer Setting** [*Patient Needs*]
		Explore ED context for using telehealth cart	**Inner Setting** [*Implementation Climate*] **Individuals** [*Knowledge and Beliefs*]
		Explore any prior experience with telemedicine HIT	**Intervention** [*Complexity*] ** Individuals** [*Knowledge and Beliefs*]
		When would this be helpful?	**Inner Setting** [*Implementation Climate*]** Individuals** [*Knowledge and Beliefs*] **Intervention** [*Relative Advantage*]
		What would make it difficult?	**Intervention** [*Complexity*]
**Act Three**	*Explore relevance, communication strategies and clinical pearls*	Ensure shared mental model of septic shock	**Outer Setting** [*Patient Needs*]
		Telehealth interactions: telephone vs video monitoring	**Intervention** [*Complexity*]
		Point-of-contact? (MD vs RN)	**Inner Setting** [*Networks and Communications*]** Intervention** [*Relative Advantage*]
		Communication strategies—in front of patient and/or families?	**Inner Setting** [*Culture*] ** Individual** [*Knowledge and Beliefs*]
		Conflicting views—how to address (TeamSTEPPS tools)	**Inner Setting** [*Culture*]**Individual** [*Knowledge and Beliefs*]
		The Sepsis Hospital Concept (eICU capabilities and limits)	**Intervention** [*Relative Advantage*]
		Wrap up: balancing barriers vs benefits	**Intervention** [*Relative Advantage, Complexity*] **Process** [*Reflect and Evaluate*]

Lists goals, outlines descriptions, and corresponding CFIR domain for each debriefing ACT within the sepsis telehealth in situ simulation

### Additional materials

Prior to the simulations, all staff were invited via email to participate in eLearning modules that reviewed the sepsis bundle components and recent system changes to the healthcare system’s automated electronic health record (EHR) sepsis best practice alert (BPA) process. The updated BPA incorporated real-time data inputs from available labs and vitals into detection algorithms for potential sepsis. Communication expectations and limitations of telehealth participation were also included in the pre-learning material.

### Simulation deployment

The 3-stage sepsis scenario was performed in situ in the two targeted rural EDs over a 1 month period. Simulations were targeted to the interprofessional teams staffing the system EDs (physician, nurse, and patient care technician). Sessions were led by emergency medicine physicians who were also experienced simulation facilitators with formal training in debriefing.

Participants were either paid for extra time to come in at the beginning or stay at the end of shift (most cases) or were relieved during their shift by paid additional staff. Simulations were performed early in the morning to catch night shift staffers at the end of their shifts, then for day shift, and staying into the late morning and early afternoon for middle shift employees. Interprofessional teams typically included one physician provider and 3–5 ED nurses and/or technicians. Nursing participation was emphasized as primary communication between the ED and the eICU occurs at the nursing level within the system.

On-duty eICU nurses participated in the actual scenario via the telehealth care mechanism, and telehealth support personnel were on site for the in situ simulations, primarily participating in the demonstration of HIT functionality in Act 1. A detailed description of the technical aspects of executing these simulations across remote sites has been described [28].

### Data collection and analysis

Pre-simulation and post-simulation surveys were collected electronically during the in situ event using tablet computers and survey software (Qualtrics© 2015, Provo, UT). Five-point (Strongly Disagree to Strongly Agree) Likert scale questions, whose content aligned with the CFIR constructs targeted in the simulation, were selected from a previously validated telemedicine readiness assessment [29] and modified to reflect the HIT under evaluation (Additional files 1 and 2). All statistical analyses were performed using the open source statistical program R (version 3.6.1) using a 2-sided alternative hypothesis with a 95% confidence level. Univariate comparisons between participants’ Likert scale ratings on the pre- and post-surveys were analyzed using paired samples *t* tests at an item level both stratified by ED and combined. Additionally, a linear mixed effects model was used to compare rating between the pre- and post-time frames while controlling for the effect of the ED site and the interaction between time frames. Participant and survey item were also included as random effects to account for variance due to repeated measurements of the same subject and item.

## Results

### Participation

A total of 60 clinicians participated in the in situ simulations across both sites. One hundred percent of participants from site 1 and 73% from site 2 availed themselves of the pre-learning opportunity. All participants consented to participate in the surveys, which were not required to participate in the education. Enrollment targets of 80% were met for participation at both sites based on nursing participation. On average, the 3-Act-3 Debrief sepsis scenario was completed in 46 min.

### Survey results

**Fig. 4:**
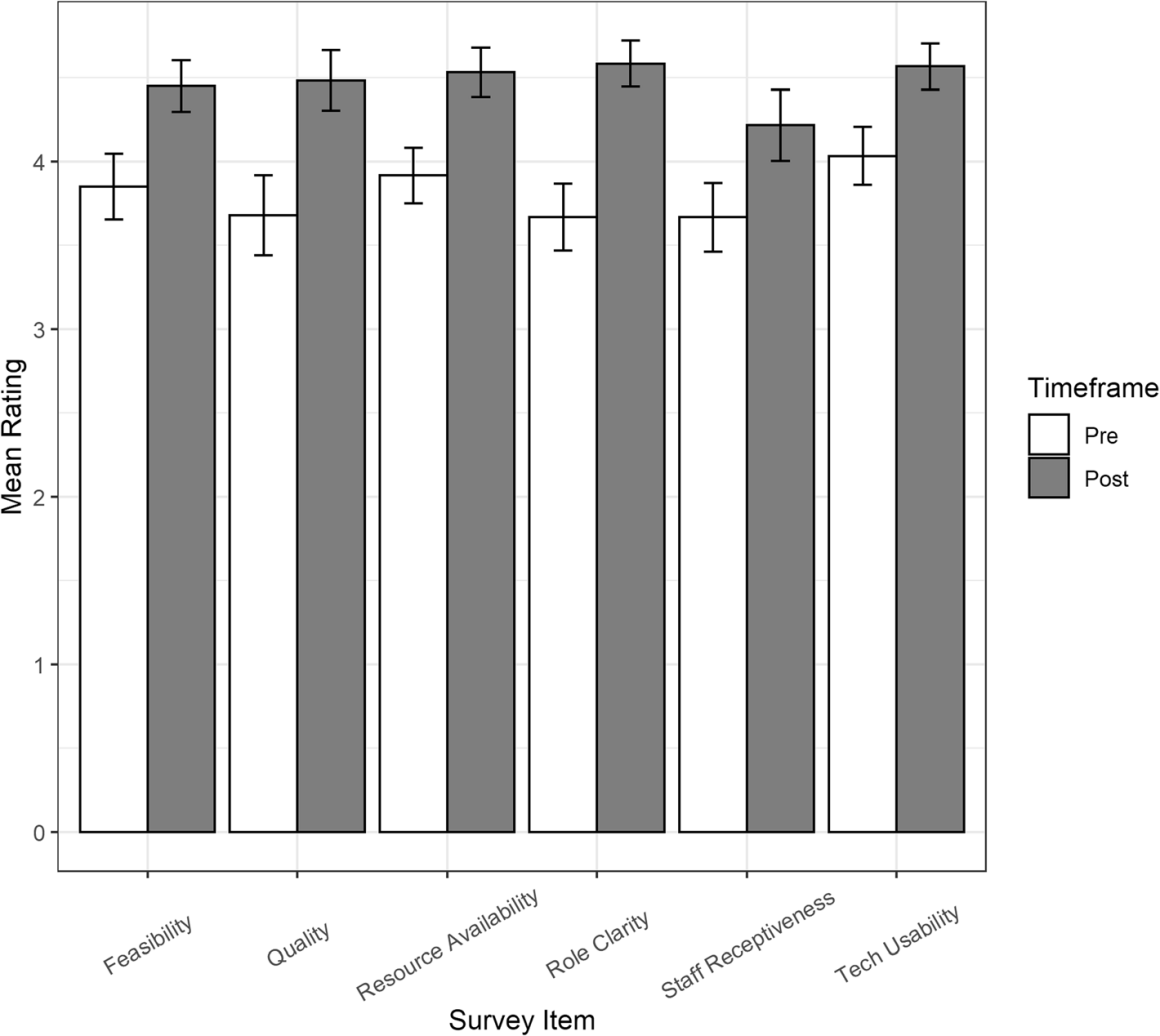
Pre–post-readiness for change results. Pre–post-simulation survey results indicating a positive shift in staff readiness for change within constructs: feasibility, quality, resource availability, role clarity, staff receptiveness, and tech usability. *N* = 58 responses for feasibility and quality. *N* = 59 responses for resource availability, role clarity, staff receptiveness, and tech usability.

**Fig. 5:**
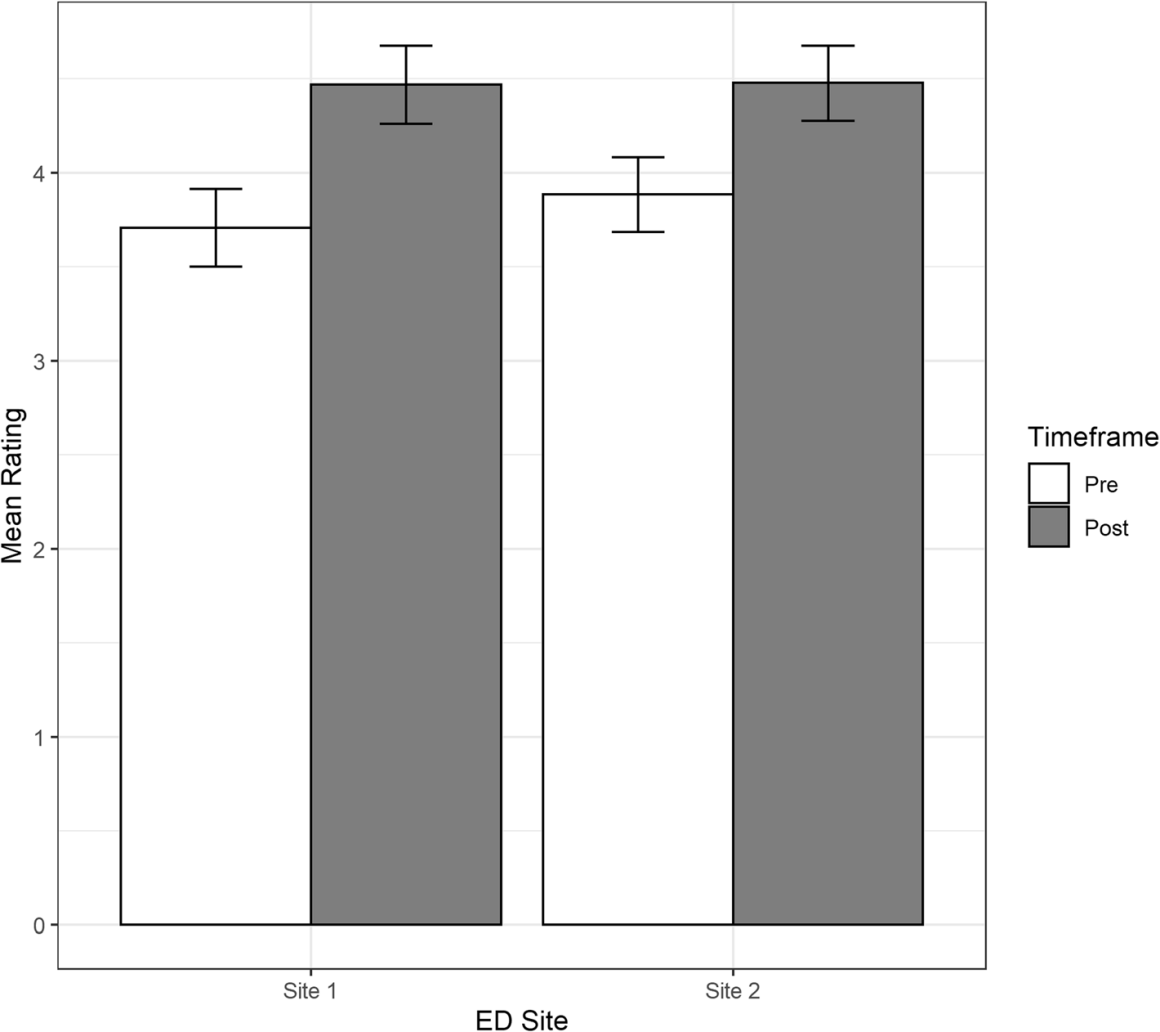
Pre- and post-survey ratings by site. A linear mixed effects model found the post-simulation timeframe was associated with significantly higher ratings (*b* = 0.76, SE = 0.11, *p* < 0.001) than the pre-simulation time frame at either site. There was also a significant negative interaction between site and timeframe (*b* = − 0.17, SE = 0.08, 0.04), which indicates that the ratings increased less from pre to post at site 2 compared to site 1. Although both sites had similar average post-ratings of approximately 4.5, site 2 had a higher mean pre-rating of 3.88 ± 0.67 which increase by 0.6 points from pre to post while site 1 had a lower mean pre-score of 3.71 ± 0.48, which increased by 0.76 points from pre to post

There were 59 survey respondents from the two sites. Ratings for the Likert-type items, both individually and mean, increased significantly from pre to post when the data was stratified by site and when it was combined (Fig. 4). Paired sample *t* tests found ratings were significantly higher on the post survey for all items and when ratings were averaged across items within a participant. The mean overall rating increased significantly from a pre-survey score of 3.82 ± 0.56 to a post-survey score of 4.5 ± 0.48, *t*(56) = 10.52, *p* < 0.001. The electronic survey did not force validate responses resulting in one participant from site 1 not rating HIT quality of care and one participant from site 2 not rating HIT feasibility. When a linear mixed model was used to control for site and account for the variance due to participant and survey item, the results indicated that both sites increased significantly, although the ratings increased less from pre to post at site 2 (Fig. 5).

## Discussion

Elements of the CFIR were successfully integrated into this in situ simulation-based introduction of a new healthcare technology by using the 3-Act-3-Debrief model to deliberately address specific CFIR constructs in a clinically relevant context. This approach differs significantly from what has traditionally been observed for the introduction of new equipment. These sessions generally take place pre-shift, adjacent to direct patient care areas, and provide supporting background knowledge with brief hands-on practice using the tool. In contrast, the approach described in this report embeds introduction of a new technology within natural workflow through an in situ simulation, thereby respecting the prior experiences of the clinical staff as adult learners who prioritize relevance to their current context and value opportunities to reflect [30].

By structuring the simulation to span the full workflow required to engage the new technology, the structural contexts surrounding its use can be more fully explored. For physician-dependent HIT, such as the electronic health record (EHR), accounting for extrinsic barriers to implementation has been recommended [31] including confidence in related organizational infrastructure, technological assistance and training, which have been correlated with clinician intent to use telemedicine-related HIT [32].

In addition, engaging interprofessional teams in our sepsis-HIT simulation provided opportunity for all debriefings to address implementation concerns in a social context framed as learning conversations. Negative comments were allowed, and, in fact, encouraged by specifically asking for barriers. In this way, key team members had the opportunity to acknowledge concerns, and issues could be addressed prior to implementation.

The survey results immediately post-simulation provided a small but favorable glimpse into the CFIR constructs in the context of caring for a simulated sepsis patient with telehealth as a proposed adjunct. It is possible that site 2 ratings increased less from pre to post than site 1 because site 2 started with higher pre-ratings, and both groups ran into somewhat of a ceiling effect on the post-survey, since ratings were made on a 5-point scale and both sites had mean post-scores of approximately 4.5. As previously reported [28], the retrospective pre–post-mean ± SD self-efficacy (11-point scale, anchored 0-100%) in the use of the telehealth cart also increased significantly, from 5.3 ± 2.9 to 8.9 ± 1.1 (Δ3.5, *p* < 0.05). The significant increases in all of these CFIR construct measures following the simulation-based intervention suggest that simulation-based introductions to HIT can positively impact individual perceptions around HIT implementation.

The intentional division of a simulated patient’s clinical course into three stages of simulation and debrief, regardless of learner actions, has also been recently described in the literature for a simulation targeting skill acquisition by medical students [33]. Similar to our study, authors utilized a 3-stage approach with a learning conversation style of debriefing. For those novice learners, the perceived learning experience was equivalent to standard post-simulation debriefing. While these two studies are promising, additional studies are necessary to further characterize the optimal use of this approach.

### Limitations

This simulation-based intervention was not intended to address all implementation considerations outlined within the CFIR. It would be impossible to cover all aspects of an implementation within a simulation of this nature, so not all the factors influencing implementation were addressed. However, this approach addressed many more facets than the traditional in-service that simply demonstrates the technology. Comparison to the usual in-service approach is limited due to lack of systematic data collection after those events.

The guided questions of the debriefing, while somewhat directive, helped bring out concerns of staff that were anticipated by the implementation group. While a fully open-ended approach to debriefing may have reduced biases in perception, time constraints forced the choice of the more guided approach while still addressing barriers and enablers. The use of physician-investigator facilitators may have limited candid sharing of negative perceptions if there was a perceived power gradient despite course debriefers leaving the room for the survey.

The ultimate success of any HIT implementation relies upon elements of the CFIR being addressed during the implementation and early adoption phase. The scope of the analysis presented here is limited in that it precedes actual implementation. The positive shifts in perceptions are necessary and valuable, but the degree to which actual adoption occurs involves more factors than could be addressed through these simulation events and is the focus of future study. Qualitative analysis of the debriefings, post-implementation focus groups, and interviews is ongoing and will be presented in future publications as will the impact of this HIT intervention on patient care outcomes.

## Conclusion

Adoption of new health information technology (HIT) may be positively impacted through leveraging in situ simulation to introduce the new platforms. The 3-Act-3-Debrief structure allows for incorporation of key constructs from the Consolidated Framework for Implementation Research (CFIR) that are correlated with successful healthcare implementations. Participation in such a structured in situ simulation around the use of a telehealth cart to improve sepsis care in rural EDs positively impacted selected CFIR measures predictive of subsequent technology adoption.

## Supplementary information

**Additional file 1.** Pre-survey. Survey questions administered to the rural ED team before the insitu sepsis simulation.

**Additional file 2.** Post-survey. Survey questions administered to the rural ED team after the in situ sepsis simulation.

## Data Availability

De-identified survey data generated or analyzed during this study are available from the corresponding author on reasonable request.

## Abbreviations

BPA: Best practice alert
CFIR: Consolidated Framework in Implementation Research
CMS: Centers for Medicare and Medicaid Services
ED: Emergency department
eICU: Electronic intensive care unit
EHR: Electronic health record
HIT: Health information technology
SP: Standardized patient
