# Supplementary material for: A novel in situ simulation framework for introduction of a new technology: the 3-Act-3-Debrief model
Source: Adv Simul (Lond). 2020 Sep 25;5:25. doi: 10.1186/s41077-020-00145-x (PMC7519488; doi:10.1186/s41077-020-00145-x)
Supplement: Supplementary file 1 — Additional file 1. Pre-survey. Survey questions administered to the rural ED team before the insitu sepsis simulation. [file 41077_2020_145_MOESM1_ESM.docx]

**Additional File 1. Pre-Survey.** Survey questions administered to the rural ED team before the in situ sepsis simulation.

**ED-Telehealth Pre-Survey**

**Demographics:**

**Subject ID**: ______________________________________

**Nurse or Physician or Mid-level or ED Technician (check one):**

□ **ED Technicians:**

□ EMT Basic □ Nursing Student with CNA License

□ **Nurses:**

□ I am an LPN □ I am an RN □ I am a BSN □ Other _____________________ (fill in)

□ **Physicians:**

□ I am board certified in Emergency Medicine

□ I am board-eligible in Emergency Medicine

□ I am board certified in Family Medicine

□ I am a PGY-3

□ **Advanced Practice Provider:**

□ I am a PA □ I am an Advanced Practice Nurse (APN)

**Years of ED Experience (for physicians include time in residency:**

□ 1-5 years

□ 6-10 years

□ >10 years

**Do you work full-time or part-time in this ED:**

□ Full time □ Part time

**Experience with Telemedicine (includes both telephone calls with telemedicine team and video-based telemedicine):**

**I use telemedicine in this ED:**

□ Frequently

□ Occasionally

□ Rarely

□ Never

**In other past jobs, ED or non-ED, I used telemedicine:**

□ Frequently

□ Occasionally

□ Rarely

□ Never

**I have used telemedicine in the care of a patient with sepsis in the ED in the past:**

□ No □ yes

**I have used telemedicine in the care of a patient with sepsis outside of the ED (i.e. the ICU, wards, etc):**

□ No □ yes

**I believe that using telemedicine will enable me to provide better quality care for patients with sepsis**.

□ Strongly Disagree □ Disagree □ Neither agree or disagree □ Agree □ Strongly agree

**Utilization during ED care of telemedicine is feasible at our hospital ED.**

□ Strongly Disagree □ Disagree □ Neither agree or disagree □ Agree □ Strongly agree

**I am clear about my roles and responsibilities as they relate to telemedicine in the treatment of ED patients with severe sepsis and septic shock.**

□ Strongly Disagree □ Disagree □ Neither agree or disagree □ Agree □ Strongly agree

**Learning to operate telemedicine technology will be easy for me.**

□ Strongly Disagree □ Disagree □ Neither agree or disagree □ Agree □ Strongly agree

**We have the resources to implement telemedicine in ED sepsis patients effectively.**

□ Strongly Disagree □ Disagree □ Neither agree or disagree □ Agree □ Strongly agree

**I believe that the ED staff is receptive to using telemedicine in the treatment of ED patients with severe sepsis and septic shock.**

□ Strongly Disagree □ Disagree □ Neither agree or disagree □ Agree □ Strongly agree

Reference:

Zapka, J., K. Simpson, L. Hiott, et al. "A Mixed Methods Descriptive Investigation of Readiness to Change in Rural Hospitals Participating in a Tele-Critical Care Intervention." BMC health services research 2013;13:33.
