# Supplementary material for: A novel in situ simulation framework for introduction of a new technology: the 3-Act-3-Debrief model
Source: Adv Simul (Lond). 2020 Sep 25;5:25. doi: 10.1186/s41077-020-00145-x (PMC7519488; doi:10.1186/s41077-020-00145-x)
Supplement: Supplementary file 2 — Additional file 2. Post-survey. Survey questions administered to the rural ED team after the in situ sepsis simulation. [file 41077_2020_145_MOESM2_ESM.docx]

**Additional File 2. Post-survey.** Survey questions administered to the rural ED team after the in situ sepsis simulation.

**ED-Telehealth Post-Survey**

**Subject ID:** _____________________________________

**Simulation Experience:**

**Please indicate your percent confidence level in managing ED patients with sepsis BEFORE and AFTER your participation in the in situ simulation session.**

|  | 0%  Not at all Confident | 10% | 20% | 30% | 40% | 50% Somewhat Confident | 60% | 70% | 80% | 90% | 100% Completely Confident |
| --- | --- | --- | --- | --- | --- | --- | --- | --- | --- | --- | --- |
| **BEFORE Session** |  |  |  |  |  |  |  |  |  |  |  |
| **AFTER Session** |  |  |  |  |  |  |  |  |  |  |  |

**Please indicate your percent confidence level in using telemedicine BEFORE and AFTER your participation in the in situ simulation session.**

|  | 0%  Not at all Confident | 10% | 20% | 30% | 40% | 50% Somewhat Confident | 60% | 70% | 80% | 90% | 100% Completely Confident |
| --- | --- | --- | --- | --- | --- | --- | --- | --- | --- | --- | --- |
| **BEFORE Session** |  |  |  |  |  |  |  |  |  |  |  |
| **AFTER Session** |  |  |  |  |  |  |  |  |  |  |  |

**Please rate the relevance of this session to your current clinical role.**

□ Extremely Irrelevant / Detrimental

□ Consistently Irrelevant / Very Poor

□ Mostly Irrelevant/ Poor

□ Somewhat Relevant / Average

□ Mostly Relevant/ Good

□ Consistently Relevant/ Very Good

□ Extremely Relevant / Outstanding

**I believe that using telemedicine will enable me to provide better quality care for patients with sepsis.**

□ Strongly Disagree

□ Disagree

□ Neither agree or disagree

□ Agree

□ Strongly Agree

**Utilization during ED care of telemedicine is feasible at our hospital ED.**

□ Strongly Disagree

□ Disagree

□ Neither agree or disagree

□ Agree

□ Strongly Agree

**I am clear about my roles and responsibilities as they relate to the use of telemedicine in the treatment of ED patients with severe sepsis and septic shock.**

□ Strongly Disagree

□ Disagree

□ Neither agree or disagree

□ Agree

□ Strongly Agree

**Learning to operate telemedicine technology will be easy for me.**

□ Strongly Disagree

□ Disagree

□ Neither agree or disagree

□ Agree

□ Strongly Agree

**We have the resources to implement telemedicine in ED sepsis patients effectively.**

□ Strongly Disagree

□ Disagree

□ Neither agree or disagree

□ Agree

□ Strongly Agree

**I believe that the ED staff is receptive to using telemedicine in the treatment of ED patients with severe sepsis and septic shock.**

□ Strongly Disagree

□ Disagree

□ Neither agree or disagree

□ Agree

□ Strongly Agree

Reference:

Zapka, J., K. Simpson, L. Hiott, et al. "A Mixed Methods Descriptive Investigation of Readiness to Change in Rural Hospitals Participating in a Tele-Critical Care Intervention." BMC health services research 2013;13:33.
